# A theoretical framework for general design of two-materials composed diffractive fresnel lens

**DOI:** 10.1038/s41598-021-94953-4

**Published:** 2021-07-29

**Authors:** Ming-Yen Lin, Chih-Hao Chuang, Tzu-An Chou, Chien-Yu Chen

**Affiliations:** 1 3D Interaction & Display Association, Taipei, Taiwan, ROC; 2 Graduate Institute of Photonics and Optoelectronics, National Taiwan University, Taipei, 10617 Taiwan, ROC; 3 Graduate Institute of Color and Illumination Technology, National Taiwan University of Science and Technology, Taipei, 10607 Taiwan, ROC

**Keywords:** Applied optics, Optical physics, Optical techniques

## Abstract

Near 100% of diffractive efficiency for diffractive optical elements (DOEs) is one of the most required optical performances in broadband imaging applications. Of all flat DOEs, none seems to interest researchers as much as Two-Materials Composed Diffractive Fresnel Lens (TM-DFL) among the most promising flat DOEs. An approach of the near 100% of diffractive efficiency for TM-DFL once developed to determine the design rules mainly takes the advantage of numerical computation by methods of mapping and fitting. Despite a curved line of near 100% of diffractive efficiency can be generated in the Abbe and partial dispersion diagram, it is not able to analytically elaborate the relationship between two optical materials that compose the TM-DFL. Here, we present a theoretical framework, based on the fundaments of Cauchy's equation, Abbe number, partial dispersion, and the diffraction theory of Fresnel lens, for obtaining a general design formalism, so to perform the perfect material matching between two different optical materials for achieving the near 100% of diffractive efficiency for TM-DFL in the broadband imaging applications.

## Previous researches on diffractive Fresnel lens (DFL)

**Figure 1:**
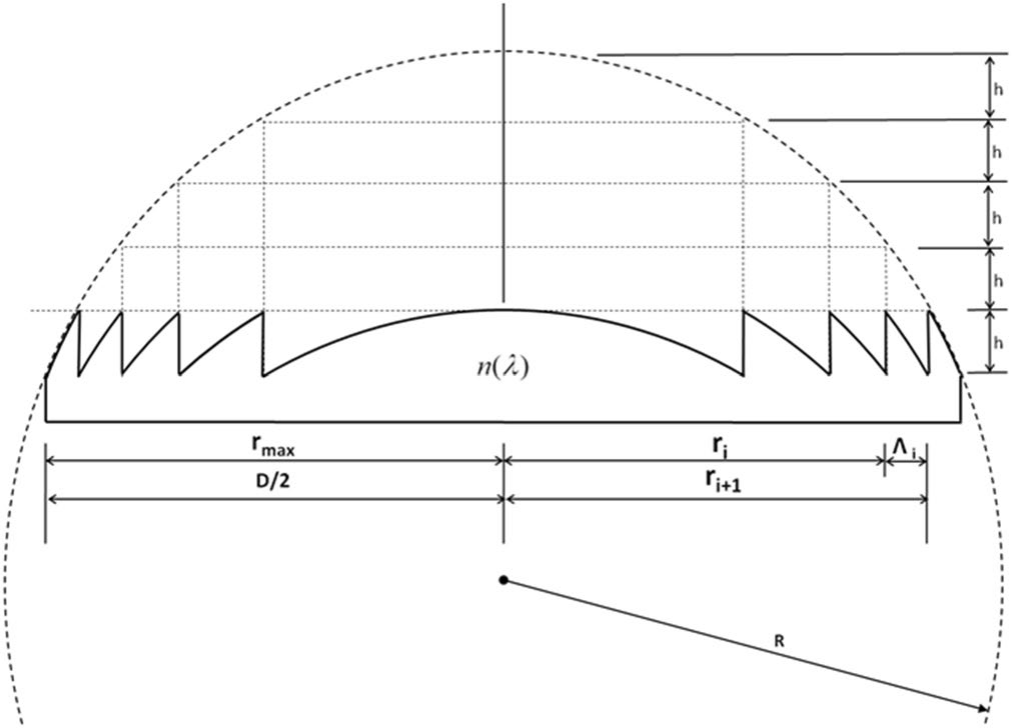
Conventional thinning process of a diffractive Fresnel lens (DFL) based on the principle of equal optical path difference.

In the thinning of optical lenses, following the principle of Equal Optical Path Difference, the surface relief of a spherical lens with radius R and refractive index n(λ) is processed to construct a Diffractive Fresnel Lens (DFL) with multiple concentric Fresnel Zone (FZ) on the surface^1–11^, as shown in Fig. 1.

Usually, exploiting the 2pπ phase (where p is a positive integer) as the Equal Optical Path Difference is the way to configure a diffractive Fresnel lens (DFL)^12–18^. The so-called DFL is generally referred to p = 1. According to the basic diffractive optics theory of DFL presented by Dale A. Buralli^12^, as shown in Fig. 1, the radial FZ structure possesses the following relations.

1 $${r}_{i}^{2}=2\mathrm{i}{\lambda }_{0}{f}_{0}$$

2 $${\Lambda }_{i}=\frac{{\uplambda }_{0}{f}_{0}}{{r}_{i}}$$

3 $$h=\frac{{\lambda }_{0}}{\mathrm{n}{(\lambda }_{0})-1}$$

4 $$D=2{r}_{max}$$

where r_i_ is the i-th FZ radius, $\mathrm{f}$_0_ is the design focal length, $\uplambda$_0_ is the design wavelength, $\Lambda$_i_ is the spacing between the i-th FZ and the i + 1-th FZ, h is the FZ height, D is the FZ diameter, and r_max_ is the outermost FZ radius.

**Figure 2:**
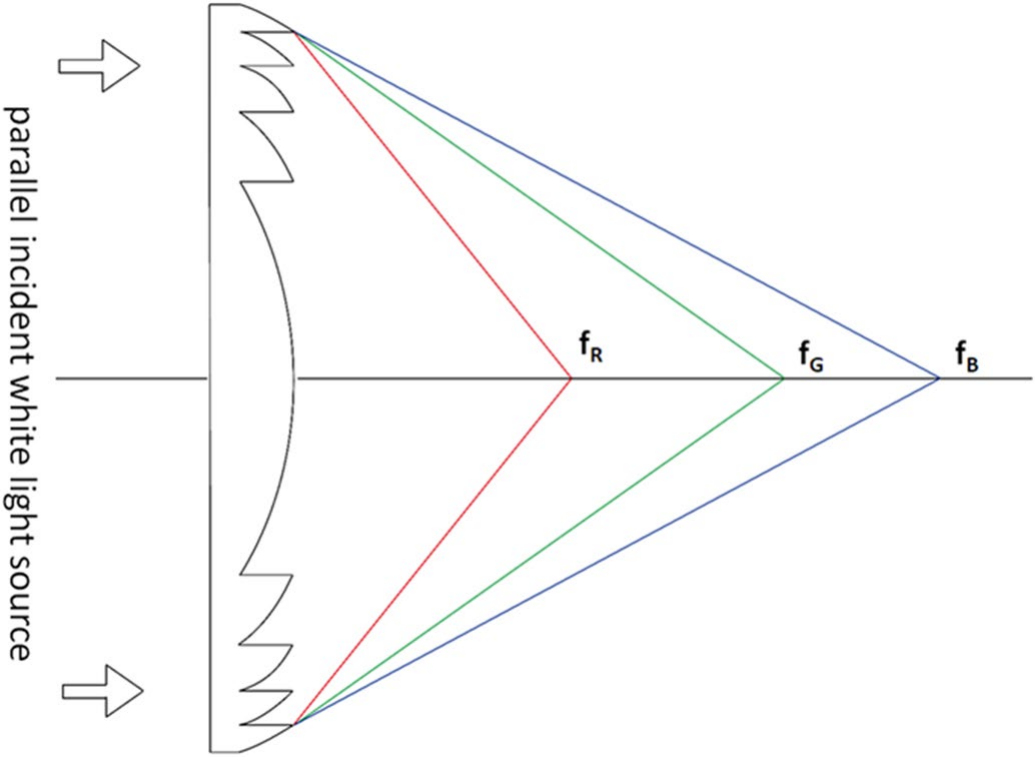
Optical power of a DFL as a function of the wavelength of the incident light.

Due to the dispersion, generally the parallel incident white light with different wavelengths, ranging from 400 to 700 nm, is focused by DFL at different focal points on the optical axis. The optical power varies linearly with the wavelength of the incident light, as shown in Fig. 2, where f_R_, f_G_, and f_B_ presenting the focal points focused by the red, green, and blue light respectively.

Furthermore, according to the above-mentioned theory of DFL^12^, the diffraction focus and efficiency of a light source with a single wavelength can be determined by the following equations.

5 $$f=\frac{{\lambda }_{0}}{\uplambda }\frac{{f}_{0}}{m}$$

6 $$\eta ={\left[\frac{\mathrm{sin}\pi (\alpha -m)}{\pi (\alpha -m)}\right]}^{2}$$

7 $$\alpha =\frac{{\lambda }_{0}}{\lambda }\frac{\mathrm{n}\left(\lambda \right)-1}{\mathrm{n}\left({\lambda }_{0}\right)-1}$$

where $\uplambda$_0_ is the design wavelength, ƒ_0_ is the design focal length, $\uplambda$ is the wavelength, f is the focal length, m is the diffracted order, η is the diffractive efficiency, α is the detuning factor, n(λ) is the refractive index of DFL as a function of wavelength λ, and n(λ_0_) is the refractive index at *λ* = λ_0_*.*

**Figure 3:**
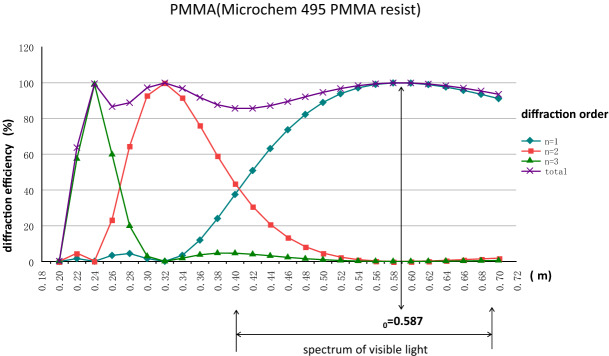
Diffractive efficiency η(λ) of PMMA as a function of the incident light wavelength λ at diffraction order m = 1, 2, and 3.

For instance, the wavelength dependent diffractive efficiency η(λ) of the optical materials of PMMA (Microchem 495 PMMA resist^19^) is calculated by Eqs. (3), (6), and (7) for the design wavelength at *λ*_0_ = 0.587 μm, FZ height at h = 1.17 μm, and the diffracted order at m = 1, 2, and 3, as shown in Fig. 3.

The calculation results reveal that most of the diffraction energy transmitted from the white incident light focused in the diffracted order at m = 1 and only a small portion of the diffraction energy contributed to the diffracted order at m = 2, 3, while the rest of the energy from the higher diffracted order being not considered because of the less contribution to the diffractive efficiency. Furthermore, in the spectrum of visible light, according to Eqs. (6) and (7), 100% diffractive efficiency can only be achieved in conditions of the diffracted order at m = 1, λ = $\uplambda$_0_ = 0.587 µm, and α = 1. Equation (6) describes how the diffractive efficiency drops off when the incident wavelength λ deviates away from the design wavelength λ_0_. More practically, the overall diffractive efficiency is generally evaluated with the mean value of spectrum distribution, as following equations.

8 $${\overline{\eta }}_{m}=\frac{1}{{\lambda }_{2}-{\lambda }_{1}}{\int }_{{\lambda }_{1}}^{{\lambda }_{2}}{\eta }_{m}(\lambda )d\lambda$$

9 $${\overline{\eta }}_{T}={\sum }_{m=1}^{\infty }{\overline{\eta }}_{m}$$

where $\overline{\eta }_{m}$ is the averaged diffractive efficiency in the diffracted order at m and $\overline{\eta }_{T}$ is the total averaged diffractive efficiency. For the parallel incident white light source, substituting the wavelength at $\uplambda$_1_ = 0.4 μm and $\uplambda$_2_ = 0.7 μm into Eqs. (8) and (9), ${\overline{\eta }}_{1}$= ~ 85.2%, ${\overline{\eta }}_{2}$= ~ 8.2%, and ${\overline{\eta }}_{3}$= ~ 1.2% are obtained in the diffracted order at m = 1, m = 2 and m = 3 respectively.

Consequently, the total diffractive efficiency from m = 1 to m = 3 comes up with ${\overline{\eta }}_{T}$ = 94.8%. In other words, there is still a certain portion of the incident light transmitted to other higher diffracted order (m > 3). For the diffracted order at m = 1 and h = 1.17 μm, about 15% of the incident light energy is disappeared. As a result, such light becomes the stray light and eventually deteriorates the imaging quality of DFL.

A solution to make up for the energy loss in the first diffracted order was first presented by Kenneth J. Weible in 1999^20^. In their research result, a blazed grating, composed of two optical materials, including glass (Schott BaF52 glass) and PC (polycarbonate), was proposed to improve the diffractive efficiency for the diffracted order at m = 1 (i.e. α = 1) by exploiting the refractive index difference Δn(λ) = n_1_(λ) − n_2_(λ) being proportional to wavelength λ. When the refractive index difference satisfies Δn(λ)/λ=constant at all wavelengths λ in the spectrum of visible light, all the incident light energy in the higher diffracted order is all transferred into the first diffracted order, as to achieve the objective of 100% diffractive efficiency. Furthermore, B. H. Kleemann mentioned the design concepts for the blazed grating composed of two optical materials in 2008^21^ and particularly named such structure as Common depth EA–DOEs. In contrast to the term “Common depth”, a singlet DFL composed of two optical materials is called Two-Materials Composed Diffractive Fresnel Lens (TM-DFL) in this study.

**Figure 4:**
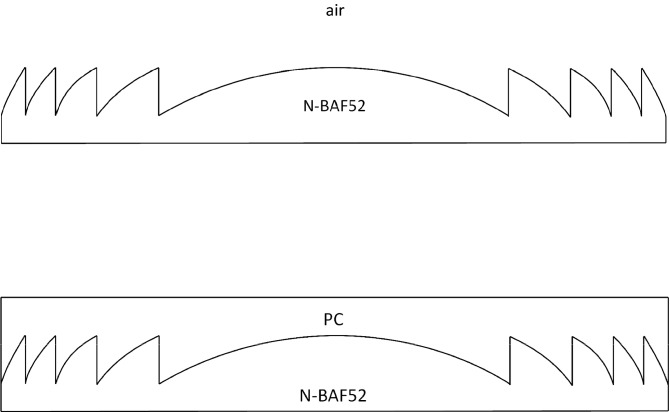
Difference between SM-DFL and TM-DFL. (**a**) material composed of a SM-DFL (**b**) material composed of a TM-DFL (**c**) diffraction efficiency of SM-DFL at m = 1 (**d**) diffraction efficiency of TM-DFL at m = 1.

According to Kenneth’s research^20^, two transparent optical materials of glass^22^ and PC^23^, as shown in Fig. 4a and b, are used for explaining the difference in the diffractive efficiency between a Single-Material Composed Diffractive Fresnel Lens (SM-DFL), i.e. the conventional DFL, and TM-DFL. For SM-FDL, the average diffractive efficiency ${\overline{\eta }}_{1}$ 84.9% is calculated by Eqs. (6), (7), and (8) for the diffracted order at m = 1 and the incident wavelength at λ = 400–700 nm, as shown in Fig. 4c. Besides, for TM-DFL, the diffractive efficiency ${\overline{\eta }}_{1}$=94.3%, as shown in Fig. 4d, is obtained by the same calculations above with a modified tuning factor α in Eq. (10) which contains the refractive index n_2_(λ) and n_2_(λ_0_) of the second material.

10 $$\alpha =\frac{{\lambda }_{0}}{\lambda }\frac{{n}_{1}\left(\lambda \right)-{n}_{2}(\lambda )}{{n}_{1}\left({\lambda }_{0}\right)-{n}_{2}({\lambda }_{0})}$$

Although TM-DFL can improve the diffractive efficiency of the conventional SM-DFL, the requirement for Δn(λ) $\propto$ λ is not perfectly satisfied. As a result, it is hard to achieve the theoretical 100% diffractive efficiency.

In fact, Andrew Wood^24^ indicated that in the case of SM-DFL composed with materials existing in nature, the detuning factor α in Eq. (7) could hardly retain the needs for α = 1 when λ deviated away from the design wavelength λ_0_. Moreover, as previously described, the regular materials, i.e. glass and PC, found by Kenneth J. Weible^20^ were able to increase the average diffractive efficiency from 85 to 94%, but it still required more effort to realize the theoretical 100% diffractive efficiency.

### Latest works on the optical design of TM-DFL: a numerical framework for the design of broadband DOEs

To overcome the above-mentioned problems, Daniel Werdehausen in 2019^25^ proposed to use dispersion-engineered nanocomposites for artificially generating a refractive index difference Δn(λ) to satisfy the requirement for $\mathrm{n}(\lambda)\propto$ λ. According to this definition^25^, the so-called nanocomposites are produced by adding a proper volume fraction of nanoparticles with a diameter smaller than 5 nm, such as Diamond, ZrO_2_, TiO_2_, ITO (indium tin oxide), and AZO (aluminum-doped zinc oxide) (presented as green stars), to the existing polymeric materials, such as PMMA (poly(methyl methacrylate)), COP (cyclic olefin copolymer), PC (polycarbonate), and PS (polystyrene) (presented as blue stars), to adjust the optical parameters of materials n_d_, v_d_, and P_g,F_. Though such new n_d_, v_d_, and P_g,F_ (presented as orange points) do not exist in nature, they can be tailored within a certain range (presented as pink area) in both the Abbe diagram and partial dispersion diagram, as respectively shown in Fig. 4a and b^25^. For instance, by adding TiO_2_ nanoparticles with different volume fraction to the polymeric material PC, the distribution of n_d_-v_d_ can extend to cover a region in 17 < v_d_ < 28 and 1.6 < n_d_ < 1.85 while the distribution of P_gF_-v_d_ covers a region in 17 < v_d_ < 28 and 0.58 < P_gF_ < 0.65.

Furthermore, the same research team in 2020^26^ proposed a design method of mapping and fitting based on the numerical computation for matching the material refractive index of TM-DFL to achieve the light diffractive efficiency higher than 99.9%. According to DOEs’ phase profiles change across the different dispersion regimes^26^, the material parameters of material 1(Mat.1) are first selected and set at n_d,1_ = 1.8, v_d,1_ = 60, and P_g,F,1_ = 0.55. By the method of mapping, the material parameters n_d,2_, v_d,2_, and P_g,F,2_ of material 2(Mat. 2) are discretely varied to calculate the distribution of the average diffractive efficiency $\overline{\eta }$ in the Abbe diagram. Similarly, the distribution of the average diffractive efficiency $\overline{\eta }$ in the partial dispersion diagram is calculated and drawn. For later discussion, the diffractive efficiency ${\overline{\eta }\left({n}_{d,2}{,v}_{d,2}\right)}_{mat1}$ and ${\overline{\eta }\left({P}_{\mathrm{g},F,2}{,v}_{d,2}\right)}_{mat1}$ are defined as a function of (n_d,2_, v_d,2_) and (P_g,F,2_, v_d,2_) respectively. The subscript Mat.1 in both function $\overline{\eta }$ stands for the values of the material parameters n_d,1_, v_d,1_, and P_g,F,1_ of Mat.1 being fixed as a constant.

The major feature of the mapping method is to discretely modulate n_d,2_, v_d,2_, P_g,F,2_ of Mat. 2 in a large region to calculate and set up both diffractive efficiency map of ${\overline{\eta }\left({n}_{d,2}{,v}_{d,2}\right)}_{mat1}$ and ${\overline{\eta }\left({P}_{\mathrm{g},F,2}{,v}_{d,2}\right)}_{mat1}$, so to find the black dotted curved lines where the calculated n_d,2_, v_d,2_, P_g,F,2_ of Mat. 2 matches to the selected n_d,1_, v_d,1_, and P_g,F,1_ of Mat.1 for achieving the near 100% diffractive efficiency. Hereafter, the black dotted curved line in ${\overline{\eta }\left({n}_{d,2}{,v}_{d,2}\right)}_{mat1}$ is named as Abbe characteristic curve of the near 100% of diffractive efficiency, or Abbe characteristic curve in brief, while the other one in ${\overline{\eta }\left({P}_{\mathrm{g},F,2}{,v}_{d,2}\right)}_{mat1}$ is named as partial dispersion characteristic curve of the near 100% of diffractive efficiency, or partial dispersion characteristic curve in brief. Finally, the most suitable material parameters n_d,2_, v_d,2_, and P_g,F,2_ can be determined by comparing the producible nanocomposites for completing the optimal match of parameters in two nanocomposite materials.

Furthermore, applying the mathematical fitting to the Abbe characteristic curve, a natural exponential function is obtained, as below.

11 $${n}_{d,2}({v}_{d,2})=a(1-{e}^{b{({v}_{d,2}-c)}^{1/3}})$$

where a = 1.839, b = 0.9919, and c = 0.8782. Note that these values are determined only for n_d,1_ = 1.8, v_d,1_ = 60, and P_g,F,1_ = 0.55. To acquire the general relationship between a, b, c, and the material parameters of MAT.1, ten of the Abbe characteristic curves with different values of n_d,1_, ranging from 1.5 to 2.0, are used to fit to obtain the n_d,1_ dependence of a, b, and c as below.

12 $$a\left({n}_{d,1}\right)=1.071{n}_{d,1}-0.07825$$

13 $$b({n}_{d,1})=\frac{0.9555}{{n}_{d,1}^{4}}-0.07825$$

14 $$c\left({n}_{d,1}\right)=53.59\sqrt{{n}_{d,1}}-16.59{n}_{d,1}-41.17$$

However, the fitting to the partial dispersion characteristic curve, is not discussed^26^.

### Major issues left by latest works

In summary, to achieve the optical parameters match for two nanocomposite materials in TM-DFL, the above-mentioned design method of mapping and fitting by the numerical computation^26^ confronts the following issues.

1. The methods of mapping can obtain a single Abbe characteristic curve and partial dispersion characteristic curve through a big volume of numerical computations at a time while losing efficiency and accuracy.
2. The fitting method can describe the Abbe characteristic curve as Eq. (11) which is a function of n_d,1_ in a special form of a natural exponential function with 1/3 power. In addition to the fitting accuracy or error, it is not a general equation for the analytical evaluation of the system. The so-called “analytical evaluation” refers to analyze the variance in the entire system caused by the parameter variation without using massive numerical computations. In other words, it is a qualitative and quantitative way to get insight into the general physical behaviors of a system by theoretical formulas.
3. Three coefficients a, b, and c of the Abbe characteristic curve merely contains n_d,1_ of Mat.1 without any further relation with v_d,1_ and P_g,F,1_ of Mat.1.
4. There is no fitting applied to the partial dispersion characteristic curve for the analytical evaluation on the relationship among parameters, i.e. (n_d,1_, v_d,1_, and P_g,F,1_) and (n_d,2_, v_d,2_, and P_g,F,2_) between two materials.

### New method: a theoretical framework for general design formalism

In contrast, we present a new method of analytical evaluation based on theoretical formulas in this study. More exactly, two characteristic curve formulas of the near 100% of diffractive efficiency are derived from the theories of Cauchy's equation, Abbe number, and partial dispersion as well as the diffractive theory of Fresnel lens. Besides, to achieve the purposes of the perfect parameters match between two optical materials in TM-DFL and the objectives of the general analyses on the optical behavior of TM-DFL**,** it completely solves the above-mentioned disadvantages of the numerical computation-based methods of mapping and fitting.

For the optical theory of DFL, the previous theoretical Eqs. (1)–(9) completely describe the relationship among the diffractive efficiency, the wavelength of the incident light, and material refractive index, where Eqs. (1)–(4) provide the design of the geometric shape of Fresnel lens, Eqs. (5)–(7) provide the focal length and diffractive efficiency after the incident light interactive with DFL, and Eqs. (8)–(9) provide the overall evaluation of diffractive efficiency.

For DFL, 2π phase shift is the necessary condition for reaching 100% diffractive efficiency which can be acquired when $\mathrm{h}\Delta \mathrm{n}({\uplambda }_{0})={\uplambda }_{0}$, where h is the FZ height, Δn(${\uplambda }_{0}$) = n(${\uplambda }_{0}$)-1 is the refractive index difference, n(${\uplambda }_{0}$) is the material refractive index of DFL at $={\uplambda }_{0}$,$\uplambda$ is the wavelength of the incident light, and λ_0_ is the design wavelength. Further, “1” in the Δn is the refractive index of air. That is, the incident light directly contacts the air after going through DFL. Since the refractive index of air is almost irrelevant to $\uplambda$, it is no way to satisfy the condition Δn($)\propto\uplambda$. Consequently, the diffractive efficiency drops off significantly when λ deviating away from λ_0_. Therefore, TM-DFL is used to effectively overcome the efficiency issue caused by the refractive index of air. For this purpose, Eq. (3) is modified as below to generate a 2π phase shift when TM-DFL is used to replace DFL.

15 $$h=\frac{{\lambda }_{0}}{{n}_{1}\left({\lambda }_{0}\right)-{n}_{2}({\lambda }_{0})}$$

where ${n}_{1}\left({\lambda }_{0}\right)$ and ${n}_{2}\left({\lambda }_{0}\right)$ are the value of two material refractive indexes in TM-DFL at $={\uplambda }_{0}$.

Moreover, the detuning factor α in Eq. (7) also needs a modification as in Eq. (10). Practically, the two optical materials used for TM-DFL have to satisfy the following requirements, including (1) optically transparent in the visible spectrum, (2) practical in mass production, and (3) Δn(λ)$\propto$. As the transparent materials existing in nature can hardly satisfy all the above conditions, especially for (3). Consequently, an optical material with an artificially tailorable refractive index, such as nanocomposite, is necessary for the realization of TM-DFL. The core of this research is to build up a theoretical foundation for the design of TM-DFL with the near 100% of diffractive efficiency by connecting theories of Cauchy's equation, Abbe number, partial dispersion, and the diffractive theory of Fresnel lens all together to derive the equation of Abbe characteristic curve and partial dispersion characteristic curve as below.

### Solution for coefficients of Cauchy's equation

In general, the refractive index n(λ) of transparent optical materials in the spectrum of visible light can be calculated by Cauchy's Eq. (16).

16 $$\mathrm{n}\left(\uplambda \right)=\mathrm{A}+\frac{\mathrm{B}}{{\uplambda }^{2}}+\frac{\mathrm{C}}{{\uplambda }^{4}}$$

where n(λ) is the refractive index depending on the light wavelength, λ is the wavelength of light in vacuum, and A, B, C are coefficients. Moreover, the dispersion of transparent optical materials can be defined with Abbe number and partial dispersion, as below.

17 $${v}_{d}=\frac{{n}_{d}-1}{{n}_{F}-{n}_{C}}$$

18 $${P}_{\mathrm{g},F}=\frac{{n}_{\mathrm{g}}-{n}_{F}}{{n}_{F}-{n}_{C}}$$

where n_d_, n_F_, n_C_, and n_g_ are the refractive indices of materials at the wavelengths of the Fraunhofer d, F, C and g spectral lines (referring to wavelength λ_d_ = 587.56 nm, λ_F_ = 486.13 nm, λ_C_ = 656.28 nm, and λ _g_ = 435.83 nm). First, n_d_, n_F_, n_C_, n_g_ and λ_d_, λ_F_, λ_C_, λ_g_ are substituted into Eq. (16) to obtain the following equations.

19 $${n}_{d}=A+\frac{B}{{\lambda }_{d}^{2}}+\frac{C}{{\lambda }_{d}^{4}}$$

20 $${n}_{F}=A+\frac{B}{{\lambda }_{F}^{2}}+\frac{C}{{\lambda }_{F}^{4}}$$

21 $${n}_{C}=A+\frac{B}{{\lambda }_{C}^{2}}+\frac{C}{{\lambda }_{C}^{4}}$$

22 $${n}_{\mathrm{g}}=A+\frac{B}{{\lambda }_{\mathrm{g}}^{2}}+\frac{C}{{\lambda }_{\mathrm{g}}^{4}}$$

Coefficients A, B, and C are solved as below.

23 $$A={n}_{d}-(\frac{B}{{\lambda }_{d}^{2}}+\frac{C}{{\lambda }_{d}^{4}})$$

24 $$B=\frac{1}{{b}_{1}{c}_{2}-{b}_{2}{c}_{1}}\frac{{n}_{d}-1}{{v}_{d}}\left({c}_{2}-{c}_{1}{P}_{\mathrm{g},F}\right)$$

25 $$C=\frac{1}{{b}_{1}{c}_{2}-{b}_{2}{c}_{1}}\frac{{n}_{d}-1}{{v}_{d}}\left[-{b}_{2}+{b}_{1}{P}_{\mathrm{g},F}\right]$$

where

26 $${\mathrm{b}}_{1}=(\frac{1}{{\uplambda }_{\mathrm{F}}^{2}}-\frac{1}{{\uplambda }_{\mathrm{C}}^{2}})$$

27 $${\mathrm{b}}_{2}=(\frac{1}{{\uplambda }_{\mathrm{g}}^{2}}-\frac{1}{{\uplambda }_{\mathrm{F}}^{2}})$$

28 $${\mathrm{c}}_{1}=(\frac{1}{{\uplambda }_{\mathrm{F}}^{4}}-\frac{1}{{\uplambda }_{\mathrm{C}}^{4}})$$

29 $${\mathrm{c}}_{2}=(\frac{1}{{\uplambda }_{\mathrm{g}}^{4}}-\frac{1}{{\uplambda }_{\mathrm{F}}^{4}})$$

Coefficients A, B, C of Cauchy's equation in Eqs. (23)–(25) clearly present the refractive index as a function of n_d_, v_d_, P_g,F_, and wavelengths of Fraunhofer d, F, C, and g spectral lines.

### Derivation of Abbe characteristic curve

As mentioned above, Eq. (6) is a general equation of diffractive efficiency while Eq. (10) defines the detuning factor α of TM-DFL which is a function of refractive index difference Δn(λ). For all wavelengths in the spectrum of visible light, the FZ height satisfying Δn(λ)h=λ is the key to direct the diffraction energy of all wavelengths toward 100% of diffractive efficiency at the diffracted order m = 1. By substituting α = 1 and λ_0_ = λ_d_ into Eqs. (10) and (15) the following equation is obtained.

30 $$\frac{{n}_{1}\left(\lambda \right)-{n}_{2}(\lambda )}{\lambda }=\frac{{n}_{1}\left({\lambda }_{d}\right)-{n}_{2}({\lambda }_{d})}{{\lambda }_{d}}=\frac{1}{h}$$

By expanding Eq. (30), it obtains

31 $${n}_{2}\left(\lambda \right)={n}_{1}\left(\lambda \right)-\frac{\lambda }{h}$$

For TW-FDL, Eqs. (30)–(31) related to the TM-DFL describe the necessary conditions to achieve the near 100% of diffractive efficiency for all wavelengths at $\uplambda =$ 400–700 nm and the diffracted order at m = 1. According to the definition in Eq. (17), let the Abbe number v_d,2_ of Mat.2 be defined as below

32 $${v}_{d,2}=\frac{{n}_{d,2}-1}{{n}_{F,2}-{n}_{c,2}}$$

where n_d,2_, n_F,2_, and n_C,2_ are the refractive indices of Mat.2 at the wavelengths of the Fraunhofer d, F, and C spectral lines. Substituting Eq. (31) into Eq. (32), it obtains

33 $${n}_{d,2}=\frac{1+({n}_{FC,1}-\frac{{\lambda }_{FC}{n}_{d,1}}{{\lambda }_{d}}){v}_{d,2}}{1-\frac{{\lambda }_{FC}}{{\lambda }_{d}}{v}_{d,2}}$$

where n_FC,1_ = n_F,1_ − n_C,1_, λ_FC_ = λ_F_ − λ_C_, and n_d,1_, n_F,1_, and n_C,1_ are the refractive indices of Mat.1 at the wavelengths of the Fraunhofer d, F, and C spectral lines. Following equation is reformed by multiplying (n_d,1_ − 1)/(n_d,1_ − 1).

34 $${n}_{FC,1}={n}_{F,1}-{n}_{C,1}=\frac{{n}_{d,1}-1}{\frac{{n}_{d,1}-1}{{n}_{F,1}-{n}_{C,1}}}=\frac{{n}_{d,1}-1}{{v}_{d,1}}$$

Substituting Eq. (34) into Eq. (33), it obtains

35 $${n}_{d,2}=\frac{1+\left(\frac{1}{{v}_{d,1}}-\frac{{\lambda }_{FC}}{{\lambda }_{d}}\right){n}_{d,1}{v}_{d,2}-\frac{{v}_{d,2}}{{v}_{d,1}}}{(\frac{1}{{v}_{d,2}}-\frac{{\lambda }_{FC}}{{\lambda }_{d}}){v}_{d,2}}$$

where v_d,1_ is the Abbe number of Mat.1. Accordingly, both formulas (33) and (35) depict the same Abbe characteristic curves for TM-DFL with the same calculated results. However, formula (35) provides a clearer scope to know how n_d,2_ is affected by n_d,1_ and v_d,1_ of Mat.1. More accurately, formula (35) can be considered as a general formula of n_d,2_ as a function of v_d,2_, n_d,1_, v_d,1_, λ _F_, λ_C_, and λ_d_. A general form of a function of n_d,2_ is defined as below.

36 $${n}_{d,2}\equiv {n}_{d,2}({n}_{d,1},{v}_{d,1},{v}_{d,2},{\lambda }_{d},{\lambda }_{F},{\lambda }_{C})$$

Unlike the conventional methods based on numerical computation, formulas (33) and (35) can accurately and immediately calculate and draw the Abbe characteristic curve in the Abbe diagram without the need for numerous numerical computations. The general behavior of n_d,2_ in Eq. (36) will be elaborated in the later discussion.

### Derivation of partial dispersion characteristic curve

According to the definition in Eq. (18), let the partial dispersion P_g,F,2_ of Mat.2 be defined as below.

37 $${P}_{\mathrm{g},F,2}=\frac{{n}_{\mathrm{g},2}-{n}_{F,2}}{{n}_{F,2}-{n}_{C,2}}$$

where n_g,2_, n_F,2_, and n_C,2_ are the refractive indices of Mat.2 at the wavelengths of the Fraunhofer g, F, and C spectral lines. Substituting Eq. (31) into Eq. (37), it obtains

38 $${P}_{\mathrm{g},\mathrm{F},2}=\frac{{n}_{FC,1}{P}_{\mathrm{g},\mathrm{F},1}-\frac{{\lambda }_{\mathrm{gF}}}{{\lambda }_{d}}{n}_{d,12}}{\frac{{n}_{d,2}-1}{{v}_{d,2}}}$$

where λ_gF_ = λ_g_ − λ_F_ and n_d,12_ = n_d,1_ − n_d,2_. Similarly, substituting Eq. (34) into Eq. (38), it obtains

39 $${P}_{\mathrm{g},\mathrm{F},2}=\frac{\frac{{n}_{d,1}-1}{{v}_{d,1}}{P}_{\mathrm{g},\mathrm{F},1}-\frac{{\lambda }_{\mathrm{gF}}}{{\lambda }_{d}}({n}_{d,1}-{n}_{d,2})}{\frac{{n}_{d,2}-1}{{v}_{d,2}}}$$

Accordingly, both formulas (38) and (39) depict the same partial dispersion characteristic curves for TM-DFL with the same calculated results. However, formula (39) provides a more clear scope to know how P_g,F,2_ is affected by n_d,1_, v_d,1_ and P_g,F,1_ of Mat.1. More accurately, formula (39) can be considered as a general formula of P_g,F,2_ since it is a function of n_d,1_, v_d,1_, P_g,F,1_, n_d,2_, v_d,2_, λ_F_, λ_g_, λ_d_. A general form of a function of P_g,F,2_ is defined as below.

40 $${P}_{\mathrm{g},F,2}\equiv {P}_{\mathrm{g},F,2}({n}_{d,1},{\mathrm{v}}_{d,1},{P}_{\mathrm{g},F,1},{n}_{d,2},{\mathrm{v}}_{d,2},{\lambda }_{d},{\lambda }_{\mathrm{g}},{\lambda }_{F})$$

Unlike the conventional methods based on numerical computation, formulas (38) and (39) can accurately and immediately calculate and draw the partial dispersion characteristic curve in the partial dispersion diagram without the need for numerous numerical computations. The general behavior of P_g,F,2_ in formula (39) will be elaborated in the later discussion.

Usually, as mentioned previously, the analytical evaluation is a way to look into the general physical behavior of a system, such as n_d2_ and P_g,F,2_ in formula (36) and (40), by applying the partial differential to the system at each parameter, such as parameters of optical materials of TM-DFL, so to understand the system response Δn_d2_ and ΔP_g,F,2_, given as below.

41 $${\Delta n}_{d,2}\cong \frac{{\partial n}_{d,2}}{{\partial n}_{d,1}}{\Delta n}_{d,1}+\frac{{\partial n}_{d,2}}{{\partial v}_{d,1}}{\Delta v}_{d,1}+\frac{{\partial n}_{d,2}}{{\partial v}_{d,2}}{\Delta v}_{d,2}$$

42 $${\Delta P}_{\mathrm{g},F,2}\cong \frac{{\partial P}_{\mathrm{g},F,2}}{{\partial n}_{d,1}}{\Delta n}_{d,1}+\frac{{\partial P}_{\mathrm{g},F,2}}{{\partial v}_{d,1}}{\Delta v}_{d,1}+\frac{{\partial P}_{\mathrm{g},F,2}}{{\partial P}_{\mathrm{g},F,1}}{\Delta P}_{\mathrm{g},F,1}+\frac{{\partial P}_{\mathrm{g},F,2}}{{\partial n}_{d,2}}{\Delta n}_{d,2}+\frac{{\partial P}_{\mathrm{g},F,2}}{{\partial v}_{d,2}}{\Delta v}_{d,2}$$

Clearly, It is no necessary to apply the same process of partial differential to both n_d2_ and P_g,F,2_ against the light wavelength λ_d_, λ_F_, λ_C_, and λ_g_ because it is no reason to change the definition of Fraunhofer line.

In summary, in contrast to the conventional method^26^, formulas (33), (35), (38), and (39) presented in this study can obtain the Abbe and partial dispersion characteristic curves of TM-DFL without numerous computations. Further, an analytical evaluation for getting more insight into the general physical behavior of TM-DFL is elaborated on below.

## Results

Hereafter, based on an example of TW-DFL^26^, we first present a quantitative result in comparison with the one obtained by the conventional method.

According to the example, the optical parameters of Mat.1 are first selected and set to n_d,1_ = 1.8, v_d,1_ = 60, and P_g,F,1_ = 0.55. Then, by the numerical computation based mapping method, the Abbe and partial dispersion diagrams are produced to generate the Abbe and partial dispersion characteristic curves. Finally, the maximum achieved diffractive efficiency can be found at η =  ~ 99.1%, n_d,2_ = 1.7, v_d,2_ = 18.4 for the Abbe characteristic curves, and η =  ~ 99.9%, v_d,2_ = 15.2, P_g,F,2_ = 0.3 for the partial dispersion characteristic curves. Meanwhile, the Abbe characteristic curve is fitted by Eq. (11) to obtain the coefficients a = 1.839, b = 0.9919, c = 0.8782.

**Figure 5:**
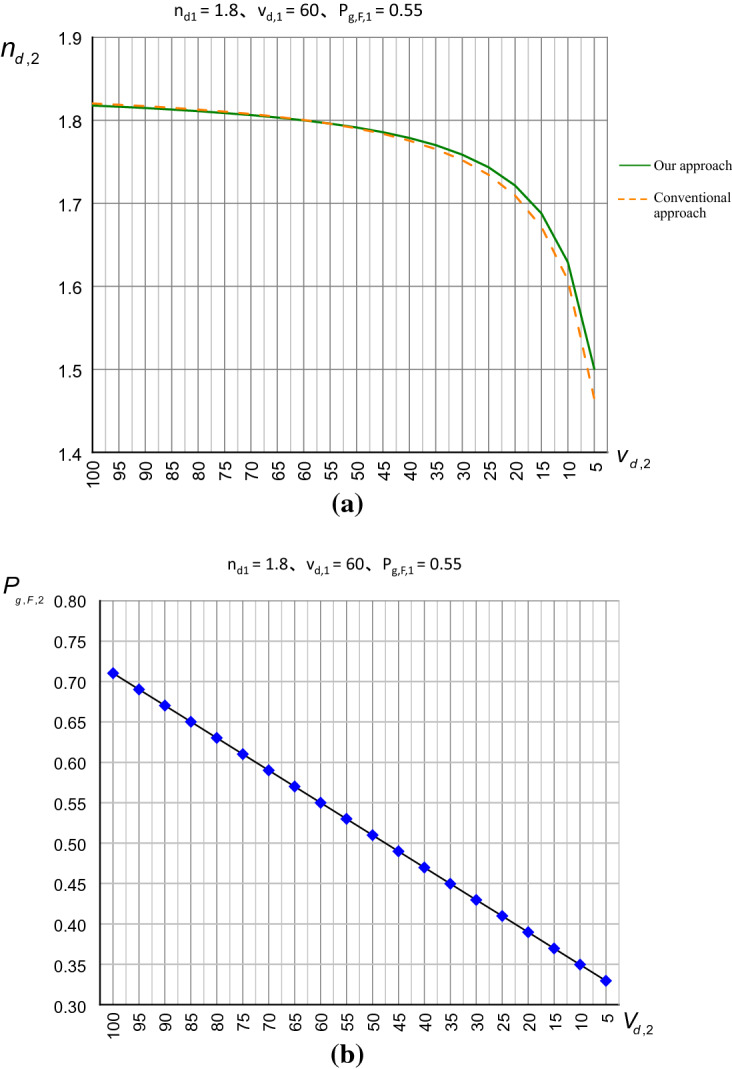
Abbe and partial dispersion characteristic curves. (**a**) our result of Abbe characteristic curve in comparison with the conventional one (**b**) our result of partial dispersion characteristic curves.

In contrast, in our studies, the Abbe and partial dispersion characteristic curves are obtained by our formulas (35) and (39) respectively, as shown in Fig. 5a and b. There are two Abbe characteristic curves on the same Abbe diagram shown in Fig. 5a, where the green solid line is our result guaranteed by the near 100% diffractive efficiency at each point on the curve while the yellow dotted line is the fitting result of above-mentioned research^26^. Apparently, the smaller v_d,2_ the larger difference shows the qualitative difference between the two methods. Also, a quantitative difference of the mean value is calculated to 0.0064. In the industrial measurement of the refractive index, this value of 0.0064 is large enough to be easily measured (note: the measurement precision of the Abbe refractometer in the market is 0.0002). In other words, the mapping and fitting methods cause non-negligible errors. Regarding the partial dispersion characteristic curve depicted on the partial dispersion diagram in Fig. 5b, it is no way to do the analytical comparison since no fitting data provided by the above-mentioned research^26^.

## Discussions

In summary, a theoretical formula-based analytical method is proposed in our studies to improve the disadvantages of the numerical computational-based mapping and fitting method. More definitely, the theory of Cauchy's equation, Abbe number, partial dispersion, and the diffractive theory of Fresnel lens are blended into optically connecting two different nanocomposite materials in TM-DFL for achieving the near 100% diffractive efficiency with all wavelengths in the visible spectrum at the first diffracted order. In addition to perfectly matching optical parameters between two materials without numerous computations, it also satisfies the objective of general analysis for TM-DFL in both quantitative and qualitative evaluations. The major features of the optical behavior of TM-DFL in our study are elaborated below.

### Feature 1: The general behavior of n_d,2_ (v_d,2_): dependent on n_d,1_ and v_d,1_ only, but independent on P_g,F,1_

**Figure 6:**
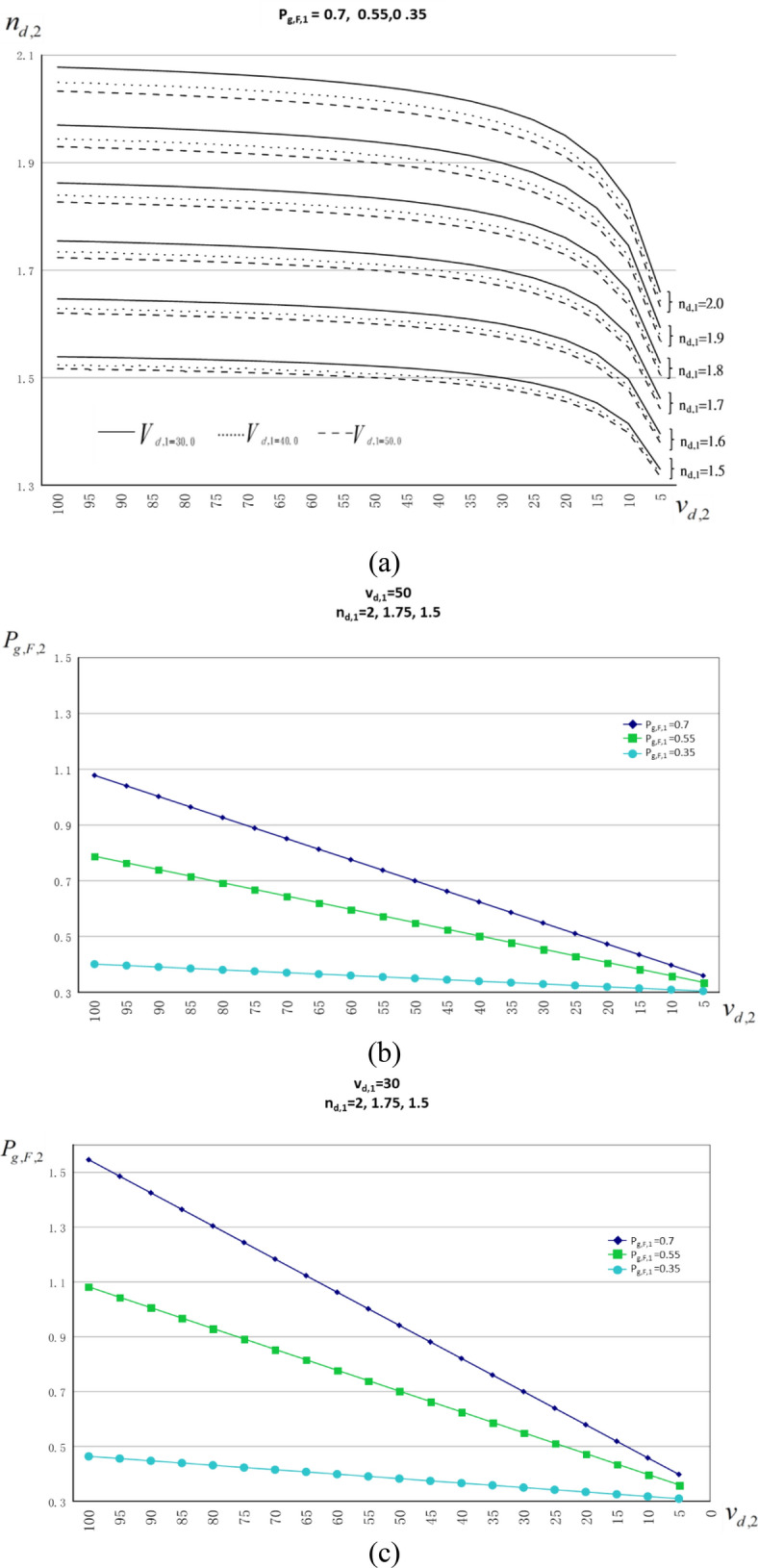
Dependence of n_d,2_ (v_d,2_) and P_g,F,2_ (v_d,2_). (**a**) n_d,2_ (v_d,2_) is dependent on n_d,1_ and v_d,1_ only but independent on P_g,F,1_, where n_d,2_ is split into a subset of lines at v_d,1_ = 30, 40, 50 when n_d,1_ is fixed. (**b**, **c**) P_g,F,2_ (v_d,2_) is dependent on P_g,F,1_ and v_d,1_ only but independent on n_d,1_ where P_g,F,2_ is constant when n_d,1_ is varied in the wide range from 2 to 1.5.

As shown in Fig. 6a, the Abbe characteristic curves of Mat.2 in the Abbe diagram is calculated by formula (35) for n_d,2_(v_d,2_) at n_d,1_ = 2.0, 1.9, 1.8, 1.7, 1.6, 1.5, and v_d,1_ = 50, 40, 30. When v_d,2_ is fixed, it shows a feature: the larger n_d,1_, the larger n_d,2_. When n_d,1_ is fixed, n_d,2_(v_d,2_) is split into a subset of lines at v_d,1_ = 30, 40, 50, for showing another feature: the less v_d,1_, the larger n_d,2_. Apparently, the Abbe characteristic curves of Mat.2 is nothing to do with P_g,F,1_ because P_g,F,1_ is not included in formula (35).

### Feature 2: the general behavior of P_g,F,2_ (v_d,2_): dependent on P_g,F,1_ and v_d,1_ only, but independent on n_d,1_

The partial dispersion characteristic curve of Mat.2 in the partial dispersion diagram is calculated by formula (39). Despite n_d,1_ being an explicit parameter in formula (39), the final calculation is irrelevant to n_d,1_. To analytically prove the independence of n_d,1_, first we need to prove ΔP_g,F,2_/Δn_d,1_ = 0 at all Δn_d,1_ and ΔP_g,F,1_ = Δv_d,1_ = Δv_d,2_ = 0, as below.

By taking the partial derivative of P_g,F,2_ in formula (39) to n_d,1_ and n_d,2_, it obtains

43 $${\Delta P}_{\mathrm{g},\mathrm{F},2}=\frac{(\frac{1}{{v}_{d,1}}{P}_{g,F,1}-\frac{{\lambda }_{gF}}{{\lambda }_{d}})}{\frac{{n}_{d,2}-1}{{v}_{d,2}}}\Delta {n}_{d,1}+\frac{(\frac{{\lambda }_{gF}}{{\lambda }_{d}}-\frac{{P}_{g,F,2}}{{v}_{d,2}})}{\frac{{n}_{d,2}-1}{{v}_{d,2}}}\Delta {n}_{d,2}$$

Since n_d,2_ is a function of n_d,1_, taking the partial derivative of n_d,2_ in formula (35) to n_d,1_, it obtains

44 $$\Delta {n}_{d,2}=\frac{\frac{1}{{v}_{d,1}}-\frac{{\lambda }_{FC}}{{\lambda }_{d}}}{\frac{1}{{v}_{d,2}}-\frac{{\lambda }_{FC}}{{\lambda }_{d}}}\Delta {n}_{d,1}$$

Substituting Eq. (44) into Eq. (43), it obtains

45 $$\Delta {P}_{g,F,2}=\frac{{v}_{d,2}}{{n}_{d,2}-1}\left[(\frac{1}{{v}_{d,1}}{P}_{g,F,1}-\frac{{\lambda }_{gF}}{{\lambda }_{d}})+(\frac{{\lambda }_{gF}}{{\lambda }_{d}}-\frac{{P}_{g,F,2}}{{v}_{d,2}})\times \frac{(\frac{1}{{v}_{d,1}}-\frac{{\lambda }_{FC}}{{\lambda }_{d}})}{(\frac{1}{{v}_{d,2}}-\frac{{\lambda }_{FC}}{{\lambda }_{d}})}\right]\Delta {n}_{d,1}$$

The sum of terms in the square brackets in Eq. (45) is always zero after completing the calculation with all parameters in the square brackets. Further, results showing the independency of n_d,1_ by the direct calculation of P_g,F,2_ (v_d,2_) in formula (39), under conditions (1) n_d,1_ = 2, 1.75, 1.5, v_d,1_ = 50, and P_g,F,1_ = 0.7, 0.55, 0.35 and (2) n_d,1_ = 2, 1.75, 1.5, v_d,1_ = 30, and P_g,F,1_ = 0.7, 0.55, 0.35, are done and shown in Fig. 6b and c respectively. Accordingly, P_g,F,2_ (v_d,2_) depends on P_g,F,1_ and v_d,1_ only, but not depend on n_d,1_.

### Feature 3: P_g,F,2_(v_d,2_) is linear, i.e. ΔP_g,F,2_/Δ v_d,2_ = constant

By taking the partial derivative of P_g,F,2_ in formula (39) to v_d,2_ and n_d,2_, it obtains

46 $$\Delta {\mathrm{P}}_{\mathrm{g},\mathrm{F},2}=\frac{{\mathrm{P}}_{\mathrm{g},\mathrm{F},2}}{{\mathrm{v}}_{\mathrm{d},2}}\Delta {\mathrm{v}}_{\mathrm{d},2}+\frac{\frac{{\uplambda }_{\mathrm{gF}}}{{\uplambda }_{\mathrm{d}}}{\mathrm{v}}_{\mathrm{d},2}}{{\mathrm{n}}_{\mathrm{d},2}({\mathrm{v}}_{\mathrm{d},2})-1}\Delta {\mathrm{n}}_{\mathrm{d},2}-\frac{{\mathrm{P}}_{\mathrm{g},\mathrm{F},2}}{{\mathrm{n}}_{\mathrm{d},2}({\mathrm{v}}_{\mathrm{d},2})-1}\Delta {\mathrm{n}}_{\mathrm{d},2}$$

Since n_d,2_ is a function of v_d,2_, taking the partial derivative of n_d,2_ in formula (35) to v_d,2_, it obtains

47 $$\frac{\Delta {n}_{d,2}}{\Delta {v}_{d,2}}=\frac{{n}_{FC,1}-\frac{{\lambda }_{FC}}{{\lambda }_{d}}({n}_{d,1}-{n}_{d,2})}{1-\frac{{\lambda }_{FC}}{{\lambda }_{d}}{v}_{d,2}}$$

Using Eq. (34) to replace n_FC,1_ in Eq. (47), then substituting Eq. (47) into Eq. (46), the local slope of P_g,F,2_ is obtained as below.

48 $$\frac{\Delta {P}_{g,F,2}}{\Delta {v}_{d,2}}=\frac{{P}_{g,F,2}}{{v}_{d,2}}+\frac{\frac{{\lambda }_{gF}}{{\lambda }_{d}}{v}_{d,2}-{P}_{g,F,2}}{{n}_{d,2}-1}\times \frac{\frac{{n}_{d,1}-1}{{v}_{d,1}}-\frac{{\lambda }_{FC}}{{\lambda }_{d}}({n}_{d,1}-{n}_{d,2})}{1-\frac{{\lambda }_{FC}}{{\lambda }_{d}}{v}_{d,2}}$$

Let’s move back to the partial dispersion characteristic curve P_g,F,2_ in Fig. 6b. In the previous discussion, we know that P_g,F,2_ depends on P_g,F,1_ and v_d,1_ only, but not depend on n_d,1_. Here, we are going to prove one more feature of the linearity in P_g,F,2_. Referring to Table 1, there are three columns, represented as P_g,F,2_(i) being a simple form used to replace the term of P_g,F,2_(v_d,2_(i)), show the data of P_g,F,2_ at different row i which is calculated by formula (39) with the related material parameters n_d,1_, v_d,1_, P_g,F,1_, n_d,2_, v_d,2_, included in formula (39). Another three columns, represented as S(i), show the data of the local slope directly calculated by Eq. (48) with the related material parameters ${n}_{d,1},{\mathrm{v}}_{d,1},{P}_{\mathrm{g},F,2},{n}_{d,2},{\mathrm{v}}_{d,2}$ included in Eq. (48). Further, more three columns, represented as ΔP_g,F,2_(i)/Δv_d,2_(i), show the local slope by directly dividing ΔP_g,F,2_(i) = P_g,F,2_(i) − P_g,F,2_(i + 1) by Δv_d,2_(i) = v_d,2_(i) − v_d,2_(i + 1). As a result, the quantitative calculations in Table 1 illustrate the linearity of P_g,F,2_ when the equality of ΔP_g,F,2_(i)/Δv_d,2_(i) = S(i) = constant is satisfied at all i = 1, 19.

**Table 1:** Equivalent results of the local slopes, i.e. ΔP_g,F,2_(i)/Δv_d,2_(i) = S(i) = constant, calculated by formula (39) and Eq. (48) show the linearity of P_g,F,2_

MAT. 1		n_d,1_ = 2.0–1.6								
		P_g,F,1_ = 50								
		V_d,1_ = 0.7			V_d,1_ = 0.55			V_d,1_ = 0.35		
i	V_d,1_(i)	P_g,F,2_(i)	ΔP_g,F,2_(i)/Δv_d,2_(i)	S(i)	P_g,F,2_(i)	ΔP_g,F,2_(i)/Δv_d,2_(i)	S(i)	P_g,F,2_(i)	ΔP_g,F,2_(i)/Δv_d,2_(i)	S(i)
**MAT. 2**										
1	100.0	1.07825	0.00757	0.00757	0.78795	0.00476	0.00476	0.40087	0.00102	0.00102
2	95.0	1.04043	0.00757	0.00757	0.76415	0.00476	0.00476	0.39578	0.00102	0.00102
3	90.0	1.00260	0.00757	0.00757	0.74036	0.00476	0.00476	0.39069	0.00102	0.00102
4	85.0	0.96478	0.00757	0.00757	0.71656	0.00476	0.00478	0.38561	0.00102	0.00102
5	80.0	0.92695	0.00757	0.00757	0.69277	0.00476	0.00476	0.38052	0.00102	0.00102
6	75.0	0.88913	0.00757	0.00757	0.66897	0.00476	0.00476	0.37543	0.00102	0.00102
7	70.0	0.85130	0.00757	0.00757	0.64518	0.00476	0.00476	0.37035	0.00102	0.00102
8	65.0	0.81348	0.00757	0.00757	0.62138	0.00476	0.00476	0.36526	0.00102	0.00102
9	60.0	0.77565	0.00757	0.00757	0.59759	0.00476	0.00476	0.38017	0.00102	0.00102
10	55.0	0.73783	0.00757	0.00757	0.67379	0.00476	0.00476	0.35609	0.00102	0.00102
11	50.0	0.70000	0.00757	0.00757	0.55000	0.00476	0.00476	0.35000	0.00102	0.00102
12	45.0	0.66217	0.00757	0.00757	0.52621	0.00476	0.00476	0.34491	0.00102	0.00102
13	40.0	0.62435	0.00757	0.00757	0.50241	0.00476	0.00476	0.33983	0.00102	0.00102
14	35.0	0.58652	0.00757	0.00757	0.47862	0.00476	0.00476	0.33474	0.00102	0.00102
15	30.0	0.54870	0.00757	0.00757	0.45482	0.00476	0.00476	0.32965	0.00102	0.00102
16	25.0	0.51087	0.00757	0.00757	0.43103	0.00476	0.00476	0.32457	0.00102	0.00102
17	20.0	0.47305	0.00757	0.00757	0.40723	0.00476	0.00476	0.31948	0.00102	0.00102
18	15.0	0.43522	0.00757	0.00757	0.38344	0.00476	0.00476	0.31439	0.00102	0.00102
19	10.0	0.39740	0.00757	0.00757	0.35964	0.00476	0.00476	0.30931	0.00102	0.00102
20	5.0	0.35957	NA	NA	0.33585	NA	NA	0.30422	NA	NA

### Feature 4: precision (error) of theoretical arithmetic

Further, let’s check out how well the material parameters n_d,2_, P_g,F,2_, v_d,2_ in Mat.2 can match up with the predetermined materials n_d,1_, P_g,F,1_, and v_d,1_ in Mat.1 to satisfy the near 100% diffractive efficiency.

According to Cauchy Eqs. (16)–(29), n_1_ ($\uplambda$) of Mat.1 and n_2_($\uplambda$) of Mat.2 can be calculated by both the predetermined n_d,1_, P_g,F,1_, v_d,1_ and the calculated n_d,2_(v_d,2_), P_g,F,2_(v_d,2_) respectively. Then, the detuning factor $\mathrm{\alpha }$($\uplambda$) is calculated by substituting n_1_($\uplambda$) and n_2_($\uplambda$) into Eq. (10). Finally, according to Eqs. (6) and (8), the diffractive efficiency ${\overline{\eta }}_{m}$ is calculated to achieve 99.95% (corresponding to the term “near 100%” used in our research) at the diffracted order m = 1 and wavelength from λ_1_ = 400 nm to λ_2_ = 700 nm. Regarding the difference in 0.05% diffractive efficiency, it is reasonable to infer that the error of 0.05% is caused by the miss of the higher approximation terms in the Cauchy Eq. (16). For the exact 100% diffractive efficiency, it can be simply obtained in the following way. Following the same treatments mentioned above, after n_1_($\uplambda$) of Mat.1 being obtained, the FZ height h is calculated first by substituting n_d,1_ and n_d,2_ into Eq. (30), then n_2_($\uplambda$) is obtained according to Eq. (31). Finally, do the same works again to get $\mathrm{\alpha }$($\uplambda$) = 1 and η(λ) = 100% at all the wavelength in the visible light spectrum.

### Feature 5: the optical behavior of convergence and divergence

**Figure 7:**
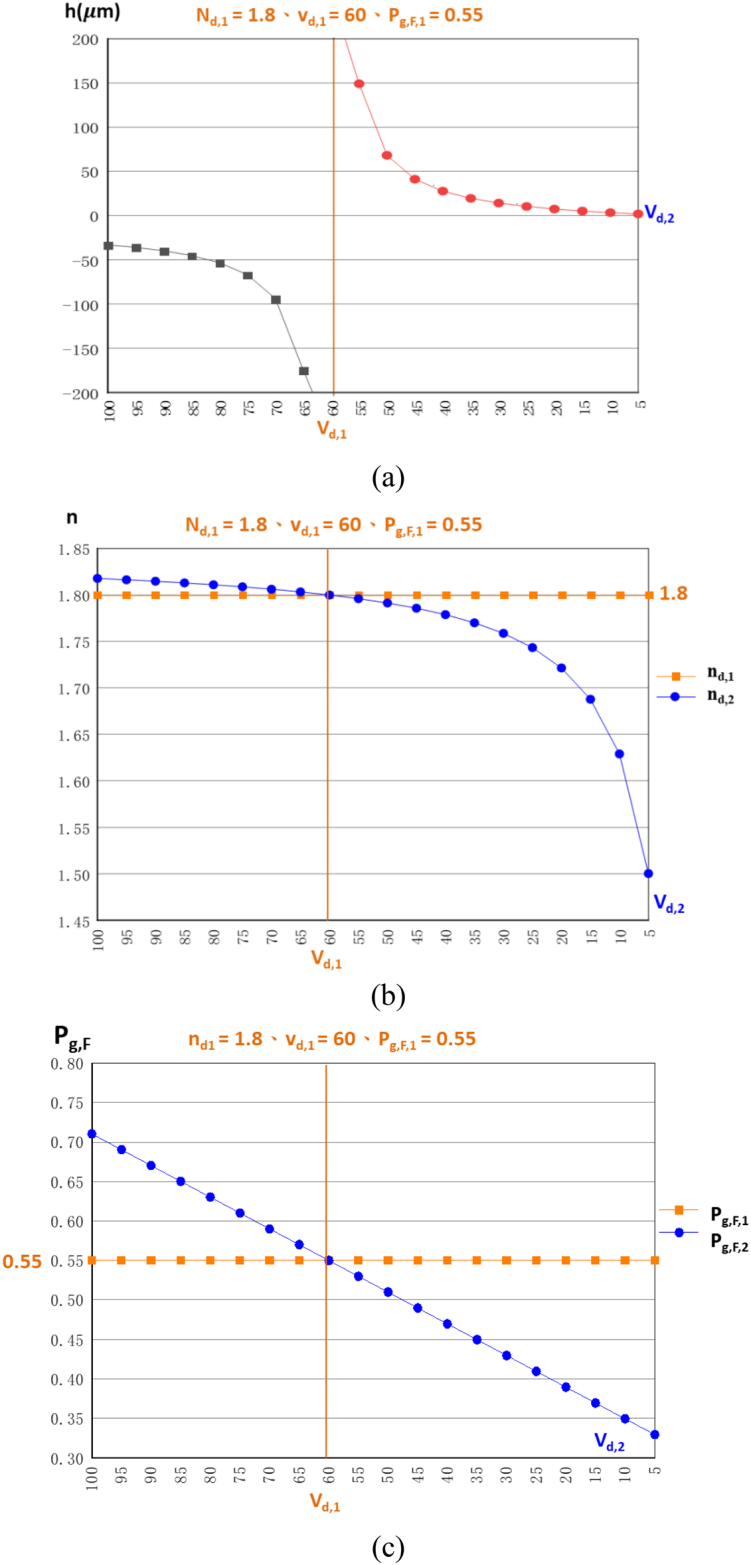
Optical behavior of convergence and divergence in TM-DFL. (**a**) FZ height h is calculated and plotted as a function of v_d.2_, where TM-DFL becomes optical transparent when v_d,2_ = v_d,1_ = 60. (**b**, **c**) TM-DFL is equipped with the focusing power when v_d,2_ < v_d,1_, h > 0, n_d,2_ < n_d,1_, and P_g,F,2_ < P_g,F,1_, while TM-DFL is equipped with the divergent power when v_d,2_ > v_d,1_, h < 0, n_d,2_ > n_d,1_, and P_g,F,2_ > P_g,F,1_.

In general, for the conventional DFL, the diffractive efficiency is determined by the FZ height h, the refractive index difference Δn, and the incident light wavelength λ while the optical focusing power is determined by the Δn and the curvature 1/R of the surface relief of DFL. Let’s take up the example used in Fig. 5a and b to further probe into the focusing power related to TM-FDL. As shown in both Figs, the Abbe characteristic curves n_d,2_(v_d.2_) and the partial dispersion characteristic curve P_g,F,2_(v_d,2_) are calculated according to formula (35) and (39) respectively. Also, following the previous treatments given in Feature 4 above, an FZ height h is plotted with the respect to v_d.2_, as shown in Fig. 7a, by employing Eq. (30) for matching h with Δn(λ) at λ = λ_d_, i.e. h(v_d,2_) = λ_d_/(n_d,1_ – n_d,2_(v_d,2_)) where n_d,1_ ≡ n_1_ (λ_d_) and n_d,2_ ≡ n_d,2_(v_d,2_), to guarantee the near 100% diffractive efficiency at the first diffracted order and all wavelengths of the incident light in the visible spectrum. Interestingly, there exists a singularity of h at v_d,2_ = 60 where v_d,2_ = v_d,1_ and n_d,2_ = n_d,1_. Consequently, the optical behavior of TM-DFL is categorized into three regions as below.

1. Transparent region: As shown in Fig. 7a, TM-DFL becomes optical transparent when v_d,2_ = v_d,1_ = 60 and n_d,2_ = n_d,1_ = 1.8. In other words, both FZ height h and focal length f_0_ approach to infinity when TM-FDL is composed of two same optical materials,
2. Focusing region: As shown in Fig. 7b and c, TM-DFL is equipped with the focusing power when v_d,2_ < v_d,1_, h > 0, n_d,2_ < n_d,1_, and P_g,F,2_ < P_g,F,1_
3. Divergent region: As shown in 7(b) and 7(c), TM-DFL is equipped with the divergent power when v_d,2_ > v_d,1_, h < 0, n_d,2_ > n_d,1_, and P_g,F,2_ > P_g,F,1_

## Conclusions

In our studies, we develop a theoretical framework to obtain a general formalism for the design of TM-DFL in broadband imaging applications. Unlike the existed approach of the numerical computation based methods of mapping and fitting, the optical theories related to Cauchy's equation, Abbe number, and partial dispersion, as well as the diffraction theory of Fresnel lens, have been perfectly blended into a new foundation for working out a TM-DFL with a precise material matching that can theoretically achieve a near 100% diffractive efficiency. The derivation of Equations for the calculations of n_d,2_(v_d,2_) and P_g,F,2_(v_d,2_) is elaborated. Also, physical behaviors of n_d,2_(v_d,2_) and P_g,F,2_(v_d,2_) are illustrated and proved, including (1) the independence of P_g,F,1_ in n_d,2_(v_d,2_), (2) the independence of n_d,1_ in P_g,F,2_(v_d,2_), (3) the linearity and constant slope of P_g,F,2_(v_d,2_), (4) beyond 0.05% of the theoretical error in the calculation of diffractive efficiency, and (5) the optical behavior of convergence and divergence. We believe that our new approach will be an effective and precise way to achieve a near 100% diffractive efficiency for the design of TM-DFL.
